# Public Hospital Pharmacists’ Perceptions and Knowledge of Antibiotic Use and Resistance: A Multicenter Survey

**DOI:** 10.3390/antibiotics9060311

**Published:** 2020-06-09

**Authors:** Kai Lun Tang, Tsyr Fen Teoh, Theng Theng Ooi, Wei Ping Khor, Sook Yee Ong, Phin Phin Lim, Sarah Abdul Karim, Sherene Su Ann Tan, Pao Pao Ch’ng, Yen Ching Choong, Weng Siang Foong, Sunitha Ganesan, Amer Hayat Khan, Long Chiau Ming

**Affiliations:** 1 Department of Pharmacy, Hospital Seberang Jaya, Ministry of Health Malaysia, Perai 13700, Malaysia; finefly37@gmail.com (T.F.T.); thengthengooi@hotmail.com (T.T.O); khor_weiping@hotmail.com (W.P.K.); sookyee_ong@hotmail.com (S.Y.O.); 2 Department of Pharmacy, Hospital Kepala Batas, Ministry of Health Malaysia, Kepala Batas 13200, Malaysia; icephin@gmail.com; 3 Department of Pharmacy, Hospital Pulau Pinang, Ministry of Health Malaysia, George Town 10990, Malaysia; sarahkarim3981@gmail.com (S.A.K.); sherenetan@live.com (S.S.A.T.); percy_1010@hotmail.com (P.P.C.); 4 Department of Pharmacy, Hospital Bukit Mertajam, Ministry of Health Malaysia, Bukit Mertajam 14000, Malaysia; celinechoong530@gmail.com; 5 Department of Pharmacy, Hospital Balik Pulau, Ministry of Health Malaysia, Balik Pulau 11000, Malaysia; wengsiangfoong@gmail.com; 6 Department of Pharmacy, Hospital Sultan Abdul Halim, Ministry of Health Malaysia, Sungai Petani 08000, Malaysia; sunitha.ganesan@yahoo.com; 7 Discipline of Clinical Pharmacy, School of Pharmaceutical Sciences, Universiti Sains Malaysia, Gelugor 11800, Penang, Malaysia; dramer2006@gmail.com; 8 Pengiran Anak Puteri Rashidah Sa’adatul Bolkiah (PAPRSB), Institute of Health Sciences, Universiti Brunei Darussalam, Gadong BE1410, Brunei Darussalam

**Keywords:** practice and perceptions, knowledge, antibiotic use and resistance, hospital pharmacists

## Abstract

Antimicrobial Stewardship Program (ASP) has been implemented in major public hospitals in Malaysia, with pharmacists playing a key role in ensuring the appropriate use of antibiotics. This survey aimed to assess the practices, perceptions, and knowledge of public hospital pharmacists on antibiotic use and resistance. A cross-sectional survey involving pharmacists from six public hospitals in Penang was conducted using a self-administered validated questionnaire. The majority of pharmacists perceived that polypharmacy (92%, *n* = 270) and overuse of broad-spectrum antibiotics (85%, *n* = 252) can potentially induce resistance of microorganisms and that ensuring the rational use of antibiotics is a shared responsibility between clinicians and pharmacists (94%, *n* = 278). A large majority of the pharmacists think that formal training in infectious disease should be a pre-requisite for pharmacists in ASP (93%, *n* = 273). In terms of antibiotic selection, the availability of antibiotics in hospital (81%, *n* = 234) and patient’s clinical condition (68%, *n* = 196) are more of a concern to the pharmacists. A total of 65% of the respondents (*n* = 192) demonstrated good levels of knowledge with a mean knowledge score of 10.1 out of 13 (95% CI: 9.95; 10.31). Pharmacists from the managerial level, ward pharmacy, in-patient, and medication therapy adherence clinic (MTAC) unit had better knowledge of antibiotics compared to pharmacists from other units (*p* < 0.001). Antibiotic knowledge gap had been identified among pharmacists in different work settings, and longer years of service does not warrant good antibiotic knowledge.

## 1. Introduction

The increased usage of antibiotics and inappropriate prescribing have exerted a selective pressure on clinically relevant bacteria, leading to the development of antimicrobial resistance (AMR). The AMR rate in Malaysia is reported to be on an increasing trend [1,2]. It will render the first-line antimicrobial ineffective, which leads to an overuse of second or third-line antimicrobials. The Ministry of Health Malaysia has started to implement policy and strategies to curb AMR in the public hospitals since early 2000s. The adaptation of the Malaysian National Antibiotic Guideline since 2008, with the implementation of the Antimicrobial Stewardship Program (ASP) in 2014 have augured well for the local AMR situation. The second edition of the Malaysian National Antibiotic Guideline was published in 2014, and the third National Antimicrobial Guideline was launched recently in 2019 [1]. Since the year 2014, ASP has been implemented in many of the state hospitals and primary care settings in Malaysia [1,2]. Following the implementation of ASP, it has been demonstrated that there was an overall 22–36% reduction in antimicrobial use [3]. Pharmacists, alongside with the Antimicrobial Stewardship (AMS) team consisting of infectious disease (ID) physicians, microbiologists, and ID nurses, play an important role in ASP [3,4]. Hospital pharmacists need to assume a role in AMS as part of their daily routine. Previous studies reported on the knowledge, attitude, and practice of antimicrobial use and resistance, factors affecting perception and attitudes towards antibiotic use mainly involved the general public, house officers, medical practitioners, community pharmacists, medical, and pharmacy undergraduates [5,6,7,8,9,10,11,12,13,14,15,16,17]. Insight into the pharmacists’ perspective and knowledge of AMS and AMR are essential to revise plans to augment their contribution in this field. Therefore, this cross-sectional multicenter survey was conducted among the pharmacists in Penang public hospitals to assess the practices, perceptions, and knowledge of antibiotic use and resistance.

## 2. Methods

### 2.1. Study Design

This was a multicenter cross-sectional survey using a self-administered printed questionnaire. All registered pharmacists and pre-registration pharmacists working at the public hospitals located in the state of Penang, Malaysia, were included in this survey. Based on the information given by the Penang Pharmaceutical Services Division, the total number of pharmacists practicing at the six public hospitals in Penang was 330. The six public hospitals were Penang General Hospital, Seberang Jaya Hospital, Balik Pulau Hospital, Bukit Mertajam Hospital, Kepala Batas Hospital, and Sungai Bakap Hospital. The pharmacists working in the private sectors (hospitals, health clinics), community pharmacy law enforcement unit, and academia were excluded from this survey. The pharmacists whom were on study leave or maternity leave were also excluded. The study respondents consisted of pharmacists from different pharmacy units, which included outpatient pharmacy, inpatient pharmacy, ward pharmacy, logistic, medication therapy adherence clinic (MTAC), Pharmacy Resources and Information Center (PRIC), management, and specialized units, i.e., therapeutic drug monitoring (TDM), clinical nutrition services, cytotoxic drug reconstitution (CDR), and pharmaceutical compounding/manufacturing. Printed questionnaires were distributed by one designated research coordinator from each hospital to the pharmacists that fulfilled the inclusion criteria. Questionnaires were recollected within the same day to discourage cross-referencing and discussion among respondents.

### 2.2. Study Instrument

The questionnaire was developed based on the study by Abbo et al. [18] Modifications were made to the questionnaire by referring to other similar questionnaires [6,8,12,15,19]. The sentence structure and content of most questions were changed to tailor to the hospital pharmacist instead of medical and pharmacy students. An initial version of the questionnaire was subjected to content validation. For content validity, the questionnaire was sent to three subject experts practicing in the state of Penang (One ID physician, one public hospital ID pharmacist, and a senior clinical pharmacy lecturer) for their opinion on the relevance and importance of the content. Necessary modifications were made based on the comments and suggestions given by the experts. The modified questionnaire was then trialed to eight pre-registered pharmacists in order to detect ambiguous questions, misinterpretation of items, and obscure statements. Face validation was also performed, and question structure was adjusted to enhance the comprehension without affecting the intended construct.

A pilot study was done with a group of 47 pharmacists from the general hospital of the Kedah state in Malaysia. The final questionnaire consisted of 53 items. Based on data from the pilot study, the internal reliability coefficient was tested, and an average Cronbach’s alpha value of 0.7 was obtained for each domain, i.e., perceptions section and practices section. However, Cronbach’s alpha for dichotomous scores in the knowledge section calculated using KR-20 was relatively low at 0.6. This probably was due to the presence of several more technical questions on antibiotic knowledge whereby respondents chose the wrong answers. The validated questionnaire consisted of four sections (53 items), which included six questions on demographic data (e.g., age, gender, years of practice, current work setting), 16 questions on “pharmacy practices”, 18 questions on “perceptions towards antibiotic use and resistance”, 13 questions on basic and specific “knowledge on antibiotic use and resistance”. The responses of the participants on the “perceptions towards antibiotic use and resistance” section were measured using a five-point Likert scale of agreement. A score of 1 was given to strongly agree, two to agree, three to neutral, four to disagree, and five to strongly disagree. In the “pharmacy practices” section, a five-point Likert scale of frequency was used with a score of one given to always, 2 to often, 3 to sometimes, 4 to rarely, and 5 to never. “Pharmacy practices” section consisted of seven basic general questions and six technical, clinical questions. Based on the finding from our pilot study, we categorized 13-item knowledge score into “Good” (≥ten correct answers), “Moderate” (six to nine correct answers), and “Poor” (<six correct answers).

### 2.3. Data Analysis

Data were analyzed by using SPSS v20. Descriptive analyses were employed to present the data as frequencies and percentages. The association between variables was analyzed using Pearson’s Chi-square (v2) test and the findings were reported with 95% confidence intervals (CIs). Items that were not answered by respondents were excluded from the analysis. The main outcomes measured were pharmacists’ antibiotic utilization knowledge, perceptions, and practices.

### 2.4. Ethical Approval

Ethical approval for this study was obtained from the Medical Research and Ethics Committee (MREC), Ministry of Health Malaysia.

## 3. Results

The response rate was 90.6%, with 299 questionnaires collected (294 questionnaires with complete answer in the knowledge section one with incomplete answer in the knowledge section). Four incomplete questionnaires were excluded. Out of the 295 completed questionnaires, 79% were females, and 21% were males. Mean age was 29.1 (±Standard deviation 3.94) years old, median (First quartile, third quartile) year of practice in the hospital was 3 (1.5, 7) years, and 91% of them have bachelor pharmacy degree as their highest qualification. In term of involvement in antibiotic dispensing, the majority (93%) claimed that they performed antibiotic dispensing in the hospital whereby on average per week, 36% of these pharmacists estimated encountering more than 20 prescriptions containing antibiotics, 35% estimated handling 10–20 prescriptions with antibiotics, and 29% estimated handling less than 10 prescriptions with antibiotics. The distribution of pharmacists according to their respective work settings is presented in Table 1.

### 3.1. Knowledge on Antibiotic Use and Resistance

From the total of 294 respondents, 65.3% had a good level (score more than 10) of knowledge on antibiotic use and resistance with a mean knowledge score of 10.1 out of 13 (95% CI: 9.95; 10.31), and only a small percentage (0.3%) scored below six. Pharmacists from the in-patient and ward pharmacy units (86%, *n* = 57 out of 66) had better antibiotic knowledge compared to pharmacists from other units (59%, *n* = 135 out of 228) (*p* < 0.001). The majority of the pharmacists answered the basic questions on antibiotic use and resistance correctly (Table 2), but the average percentage of correct answers was significantly lower (*p* < 0.001) for a set of specific questions on clinical use of antibiotics. More than half of the pharmacists did not get the correct answer for the clinical use of antibiotics related to surgical prophylaxis, hospital-acquired pneumonia, and understanding of pharmacokinetic/pharmacodynamic properties of antibiotics. Besides, the percentage of correct answers for specific questions related to carbapenem usage was noted to be relatively low (64%) compared to questions relating to the treatment of Methicillin-resistant *Staphylococcus aureus* (MRSA) and bacterial meningitis.

The knowledge score between different pharmacy service settings was significantly different. The results showed that more than 70% of pharmacists from the ward pharmacy unit (100%), pharmacy management unit (100%), in-patient unit (73%), and MTAC units (73%) had significantly higher knowledge score (>10). It was followed by specialized units (67%), i.e., therapeutic drug monitoring, clinical nutrition services, cytotoxic drug reconstitution and manufacturing, out-patient unit (62%), pharmacy resources and information center (42%), logistic unit (37%), and pre-registered junior pharmacist (provisionally registered pharmacist) (55%) (Table 3).

### 3.2. Perceptions of Antibiotic Use and Resistance

The majority of the pharmacists perceived that polypharmacy of antibiotics could cause unnecessary adverse effects, and overuse of broad-spectrum antibiotics may potentially increase antibiotic resistance. The respondents had mixed responses concerning the overuse of antibiotics in the hospital; many of them remained optimistic that antibiotic resistance has yet to be a significant problem. The overall pharmacists’ perceptions were positive towards AMS as the majority agreed that ensuring the rational use of antibiotics is a shared responsibility between clinicians and pharmacists, as well as the patients. The summary of the findings is presented in Table 4. 

More than 80% of hospital pharmacists agreed that the presence of the AMS program would affect antibiotic use and resistance in hospitals, whereby the ID team should be consulted at an early stage and pharmacist should be actively intervening inappropriate antibiotic use. The variability and availability of antibiotics in each hospital drug formulary was perceived to be one of the main factors affecting antibiotic use. Generally, the majority of hospital pharmacists also agreed that continual in-house audits on antibiotic use, the presence of National Antibiotic Guidelines, and the implementation of newer broad-spectrum antibiotic use restriction by pharmacists would impact antibiotic use and resistance. However, our findings indicated a mixed response concerning whether clinician’s preference for innovator antibiotics and pressure from the patients to prescribe antibiotics would have any influence on antibiotic use in the hospital (Table 5).

### 3.3. The Practice of Hospital Pharmacist Towards Antibiotic Recommendations

The variability in the types and availability of antibiotics in hospitals were the number one factors which influenced pharmacist’s recommendation to doctors on the choice of antibiotic (Table 6). Public hospital pharmacists had placed a considerably equal amount of attention to each aspect (patient, disease, and drug factor) when giving a recommendation to doctors regarding the choice of antibiotic, i.e., the severity of patient’s clinical condition (68%), risk of missing an infection (63%) and toxicity profile of the antibiotic (63%). However, only 50% of the respondents were concerned about the use of specific groups of antibiotics, which in turn may increase the risk of inducing resistant pathogens. Up to 63% of the respondents took into account the current stock level of antibiotics before offering recommendations on the choice of antibiotic. On the other hand, only a small proportion was concerned about cost savings for the hospital and whether the antibiotic is a generic or innovator product.

Before the release of the 3rd Malaysian National Antibiotic Guideline, the 2nd edition of the National Antibiotic Guideline 2014 was the primary source of information for public hospital pharmacists and the majority (>90%) of the respondents used the National Antibiotic Guideline as one of their sources of information during practice in the hospital. Second to it was the continuous medical education sessions provided by the hospital pharmacy colleagues or doctors, followed by the past clinical attachment to a ward pharmacy unit, antibiotic updates from medical journals, seminars provided by pharmaceutical companies, and international guidelines (Infectious Diseases Society of America guideline, Sanford guideline). In contrary to their positive perception towards ASP, only a small proportion (24%) of hospital pharmacists claimed that they obtained their source of information via consultation with the hospital ID team. Interestingly, pharmacists who claimed that they were exposed to the Infectious Disease Society of America and Sanford guidelines or consultations with the ID teams during practice were associated with a higher antibiotic knowledge score.

## 4. Discussion

This is the first documented study in Malaysia targeting only the pharmacists practicing in public hospitals, which include both tertiary hospitals and secondary hospitals. Overall, the current sample was appropriate to represent the hospital pharmacists who were directly or indirectly involved in antibiotic use.

The majority of hospital pharmacists demonstrated good knowledge on antibiotic use and resistance. This should enable the pharmacist working in the public hospital to educate the general public in the region as more than half of the general public in Malaysia still have inadequate knowledge of antibiotic use [9,20], especially those with a lower education level [9]. However, not all pharmacists demonstrated sound technical knowledge on clinical use of antibiotic (e.g., antibiotic for extended spectrum beta-lactamases, producing pathogens, hospital-acquired pneumonia, surgical prophylaxis, and the pharmacodynamic aspect of specific antibiotics). Unpublished data from a similar study which involved doctors from the same public hospitals in the region also showed lower percentage of correct answers for technical questions related to antibiotic use in surgical prophylaxis, extended spectrum beta-lactamases (ESBL) pathogens, hospital-acquired infection and appropriate use of vancomycin. With good technical knowledge on antibiotic use and resistance, hospital pharmacists who had direct interaction with the general public at the dispensing counters, in the pharmacist-run clinics, and in the wards could assume the role to promote prudent use of antibiotics. In spite of this, it should be highlighted that the technical knowledge reported in this study did not cover topics such as appropriate choice of empirical antibiotics for various common diseases, interpreting resistant patterns of pathogens based on laboratory cultures and sensitivity reports, identifying inappropriate combination of certain antibiotics, renal dosing for important antibiotics, and intravenous to oral conversion of certain antibiotic groups. Public health campaigns on antibiotic use and resistance covering hospitals, media, supermarkets, religious places, schools, and community halls might help to increase the knowledge of the general public, and reach out to those high-risk groups who were prone to misconceptions about antibiotic use [9,16].

The number of years of pharmacy practice in the hospital, similar to other studies [7,14,17,21], was not significantly associated with antibiotic knowledge. This probably was due to the fact that antibiotic practice guidelines change from time to time depending on the continuously evolving epidemiology of infections and the emergence of new antibiotic research findings. Hence, hospital pharmacists need to keep abreast with the latest antibiotic practice recommendations. When senior pharmacists are more involved in the managerial aspect, they may gradually lose touch with the more recent updates of antibiotic dosing and infectious disease management.

Interestingly, the knowledge gap among hospital pharmacists from different work settings was identified with pharmacists from the in-patient and ward pharmacy units who had better antibiotic knowledge compared to pharmacists from other units. This probably due to the fact that ward pharmacists and in-patient department pharmacists had more exposure to complex antibiotic use in ward throughout their work routine. This particular group of pharmacist’s acts as a good point of reference on antibiotic use and resistance. Furthermore, selected pharmacists, especially those practicing in the ward and the in-patient department, could be trained as ID pharmacists in line with a recommendation by the Infectious Disease Society of America ASP Guidelines that suggested that ASP should be administered by either the ID physician or ID pharmacist [22]. Nevertheless, the task of building the AMS team in all public hospitals remains challenging as most of the well-trained ID physicians were assigned to the large referral tertiary hospitals.

The majority of the pharmacists agreed that polypharmacy of antibiotics increased patient’s risk of adverse drug effects. Local data reported that the incidence of the adverse drug reactions among patients prescribed with antibiotics was about 1.1%, with beta-lactam antibiotics, cephalosporins, vancomycin, and trimethoprim/sulfamethoxazole topped the list as the major contributors to such incidences [23]. Most commonly reported adverse drug reactions involved the skin, gastrointestinal system, and kidney function [23]. Positive perceptions held by the hospital pharmacists towards this issue could help to prevent overuse of antibiotics in the public hospitals.

Based on the National Surveillance of Antibiotic Resistance data [2], there was an increase in cases of meropenem-resistant *Acinetobacter baumanii,* emergence of ESBL among *enterobacteriaceae*, cases of carbapenem-resistant *enterobacteriaceae*, vancomycin resistant *Enterococcus faecium*, and erythromycin-resistant *Streptococcus pneumoniae*. Although the majority of pharmacists perceived that the overuse of broad-spectrum antibiotics may potentially increase antibiotic resistance, there was a mixed response concerning the overuse of antibiotics in the public hospitals. Many of the hospital pharmacists remained optimistic that antibiotic resistance has yet to be a significant problem in hospital. This false optimism could be due to a few possibilities. First of all, the hospital pharmacists may not be aware of the information of the increasing defined daily dose and resistance pattern published in the National Antibiotic Guideline 2014. It could also be possible that hospital pharmacists are not interested in taking note of these alarming statistics. On the other hand, many of the pharmacists agreed that an antibiogram should be made available in the hospital to ensure proper selection of antibiotics. However, the availability of an antibiogram for each hospital depends on the capacity of the microbiology lab of each hospital to produce it. It is worth mentioning that the antibiogram was not available in all public hospitals.

In accordance with findings from other studies [6,19,22,24,25], majority of hospital pharmacists had positive perception towards AMS where there was high percentage of agreement that ensuring rational use of antibiotics is a shared responsibility between clinicians and pharmacists, and formal training in ID and AMS should be a pre-requisite for pharmacists to be part of the ASP. Although our national AMS policy mentioned that core members of the AMS team should be multidisciplinary, training and certification requirements for infectious diseases-trained clinical pharmacists have not been established. Most of the pharmacists appointed to join AMS team had to develop their interest in infectious diseases by self-directed learning or on-the-job experiences. This mode of training, however, is not considered feasible or sufficient for reliable training of future clinical specialists in infectious diseases [26].

The development of the AMS protocol by the Ministry of Health and implementation of AMS in public hospitals had successfully forced a downward trend in antibiotic utilization in 2016 [2]. Several measures were implemented and monitored by the pharmacist in hospital, such as the “72-h stop order for carbapenem, vancomycin, and some selected high-end antibiotics” which requires physicians to review and justify continuation of treatment with the broad-spectrum antibiotic, audit on IV Cefuroxime usage in surgical & orthopedic departments, restrictions on usage of injection Colistimethate, establishment of policy on surgical antimicrobial prophylaxis, etc. Given the support from the Ministry of Health, the pharmacist as part of the clinical support team in the hospital should serve as advisors to doctors in antibiotic decision-making and there should be mutual understanding as partners in the effort to curb the proliferation of AMR [25].

In terms of pharmacist perception towards factors affecting antibiotic use and resistance, a majority of the respondents agreed that variability and availability of antibiotics in each hospital drug formulary would affect antibiotic use. In contrast to perceptions of medical practitioners [14,17], hospital pharmacists believed that pressure from patients to prescribe antibiotics no longer have much impact on the prescriber’s decision to use an antibiotic. Furthermore, there was mixed response concerning whether the clinician’s preference for innovator antibiotics will affect antibiotic use.

Generally, the majority of hospital pharmacists also agreed that continual in-house audits on antibiotic use, presence of National Antibiotic Guideline, and implementation of broad-spectrum antibiotic use restriction by pharmacists will impact on antibiotic use and resistance. These findings were supported by other studies [4,24,27,28], whereby the hospital pharmacists considered restrictions as a reasonable method to control antibiotic use, and pharmacists assumed responsibility in providing audit and feedback regarding antibiotic use. Consistent with unpublished data which involved doctors from the same public hospitals in the region, perception of hospital pharmacists was unanimous that the presence of ASP is essential and will affect antibiotic use and resistance in hospital whereby an ID team should be consulted at an early stage. The pharmacist should be actively involved in the monitoring of inappropriate antibiotic use, especially those working at the dispensing counter, in-patient pharmacy, and in the ward treating hospitalized patients.

A recent systematic review [29] identified several factors known to affect the prescribing of antibiotics, i.e., the demands from patients, pharmaceutical company marketing activities, economic factors, availability and supply of antibiotics, instructions at organizational level to clear near-expiry antibiotics, and limited up-to-date information sources. In contrast, only a small proportion of hospital pharmacists would consider cost savings for the hospital and whether the antibiotic is an innovator or generic brand before recommending an antibiotic. The lack of sensitivity towards generic or innovator brands among public hospital pharmacists was probably due to the frequent brand switch from innovator to generic products as part of cost-saving measures. On the other hand, the availability and variability of antibiotics in hospital were checked by the pharmacists before giving recommendation on the choice of antibiotic because some district public hospitals may not have the first-line antibiotic. Hence, pharmacists need to be able to recommend a suitable alternative caused by stock-out. Pharmacists in the public hospital are held responsible for the good governance of antibiotic, especially in the control of availability of drugs and the stock level. New drugs that were proposed to be listed into the hospital formulary would need to be adequately justified by the clinician and then reviewed by the pharmacist. Besides, in general clinical practice, the pharmacist’s recommendation for antibiotics should always be based on a patient’s clinical condition, including immunocompromised status, site of infection, possible pathogens, coverage, and toxicity profile of the selected antibiotic.

Since the first publication of the Malaysian National Antibiotic Guideline in 2008, it had gradually become the primary source of reference for many public hospital pharmacists. The second edition of the National Antibiotic Guideline published in 2014 had become more favorable among pharmacists in public hospitals. Both the National Antibiotic Guideline softcopies had been made available for free online download. It is not surprising to find that only a small proportion of public hospital pharmacists claimed that they obtained their source of information via consultation with the hospital ID team. This was because a well-established ID team was not readily available in certain hospitals in the state of Penang. At the time of discussion, ID consultant is only available at Penang General Hospital. In view of the high usage of Malaysian National Antibiotic Guideline 2014 among the hospital pharmacists, it is hoped that the latest Malaysian 3rd edition National Antibiotic Guideline 2019 would be adopted as a standard practice among all healthcare providers in the public hospitals.

## 5. Conclusions

Generally, pharmacists’ perceptions were positive towards the AMS program as the majority agreed that ensuring rational use of antibiotics is a shared responsibility between clinicians and pharmacists. This study also reveals that pharmacists in public hospitals have good basic knowledge on antibiotic use and resistance. Longer years of service do not warrant good antibiotic knowledge, and the antibiotic knowledge gap had been identified among pharmacists in the different work settings. The AMS program, early ID referral, pharmacist intervention, audit on antibiotic use, restrictive policy by pharmacy departments, and timely updates of the National Antibiotic Guideline are perceived as the important factors that may affect antibiotic use and resistance in hospitals.

## Figures and Tables

**Table 1:** Distribution of respondents according to the type of pharmacy settings.

Pharmacy Services	Number of Pharmacists (*n* = 295)	Percentage %
Outpatient unit	102	34.6
Inpatient unit	33	11.2
Ward pharmacy unit	33	11.2
Logistic unit	27	9.1
Medication Therapy Adherence Clinic (MTAC)	15	5.1
Management and administration unit	10	3.4
Pre-registration pharmacist on rotation	44	15.0
Pharmacy resources and information center	12	4
Other units (TDM, CDR, manufacturing, clinical nutrition)	19	6.4

Footnote: TDM—Therapeutic Drug Monitoring unit, CDR—Cytotoxic Drug Reconstitution unit.

**Table 2:** Respondents’ knowledge on antibiotic use and resistance (*n* = 294 **).

No	Statement and Questions	Correct Answer
Respondents answered either TRUE or FALSE for each statement		Number (%)
1	Antibiotic utilization is measured in the World Health Organization defined daily doses.	253 (86) True
2	Antibiotic is prescribed as prophylactic measured to fight infection caused by multiple drug-resistance pathogens.	188 (64) False
3	Resistant bacteria cannot be easily spread in healthcare institutions and communities.	265 (90) False
4	Resistant genes can be transferred from one bacterium to another via plasmid transfer.	270 (92) True
5	Cross-resistance is the condition in which the resistance occurs to a particular antibiotic that often results in resistance to other antibiotics, usually from a similar chemical class.	279 (95) True
6	Antimicrobial resistance can be minimized by de-escalation therapy based on culture and sensitivity results.	282 (96) True
7	To curb the progression of antibiotic resistance, the two major interventions are infection control (to prevent transmission), and judicious antibiotic use.	288 (98) True
Specific questions on clinical use of antibiotic Respondents required to choose the best answer		
8	Choice of first-line antibiotic for Methicillin-Resistant *Staphylococcus aureus* (MRSA) bacteremia.	285 (97)
9	Most appropriate antibiotic to treat extended spectrum beta-lactamases (ESBL) *Klebsiella pneumonia* sepsis.	188 (64)
10	Empirical treatment of acute bacterial meningitis based on Malaysia National Antibiotic Guideline 2014.	253 (86)
11	The most appropriate antibiotic for surgical prophylaxis is in a total knee replacement surgery.	144 (49)
12	Ability to recognize the hospital-associated infection and suggest empirical treatment for hospitalized older patients diagnosed with pneumonia.	138 (47)
13	Based on antimicrobial killing properties, suggest the antibiotics that would exert maximum efficacy if extended infusion time from 30 min to 3 hours.	123 (42)

** One respondent was excluded due to incomplete answers to the knowledge section.

**Table 3:** Knowledge score breakdown according to the different pharmacy settings.

	Number of Respondents Based on Pharmacy Service Settings								
Knowledge Score	Ward Pharmacy	Management	In-Patient	MTAC	Out-Patient	Logistic	Specialized Units	PRIC	Pre-Registered Junior
<10	0	0	9	4	39	17	6	7	20
≥10	33	10	24	11	63	10	12	5	24
Total	33	10	33	15	102	27	18	12	44
Percent, %	100	100	73	73	62	37	67	42	55

Footnote: Pearson Chi-Square = 39.383, Cramer’s V value = 0.366; *p*-value < 0.001. MTAC—Medication Therapeutic Adherence Clinic; PRIC-Pharmacy Resources and Information Center.

**Table 4:** Summary of pharmacists’ perceptions of antibiotic use and resistance.

Statement on Antibiotic Use and Resistance	Strongly Agree/Agree	Strongly Disagree/Disagree/Neutral
Antibiotics are overused in my hospital	45% (*n* = 132)	55% (*n* = 162)
Antibiotic resistance is a significant problem in hospital	34% (*n* = 100)	66% (*n* = 195)
Polypharmacy of antibiotics: Unnecessary adverse effect and induce resistant microorganism	92% (*n* = 270)	8% (*n* = 24)
Over use of broad-spectrum antibiotic may increase antibiotic resistance	85% (*n* = 252)	15% (*n* = 43)
Higher potency antibiotic will be developed in future	33% (*n* = 98)	76% (*n* = 197)
Antibiogram should be made available in hospital to ensure proper antibiotic selection	82% (*n* = 241)	18% (*n* = 54)
Ensuring rational use of antibiotics is a shared responsibility between clinician and pharmacist	94% (*n* = 278)	6% (*n* = 17)
Formal training in ID or AMS should be a prerequisite for pharmacist in order to be part of the ASP	93% (*n* = 273)	7% (*n* = 22)

Footnote: Item not answered by respondents was excluded for analysis. ID—Infectious disease, AMS—Antimicrobial Stewardship, ASP—Antimicrobial Stewardship Program.

**Table 5:** Perceptions towards factors affecting antibiotic use and resistance in the hospital.

Factors Affecting Antibiotic Use and Resistance in Hospital	Strongly Agree/Agree	Strongly Disagree/Disagree/Neutral
Presence of ASP	90% (*n* = 266)	10% (*n* = 29)
Early referral to ID team	81% (*n* = 238)	19% (*n* = 57)
Intervention by hospital pharmacist	80% (*n* = 236)	20% (*n* = 58)
Variability & availability of antibiotics in hospital	80% (*n* = 235)	20% (*n* = 58)
Continuous in-house audit on antibiotic use	79% (*n* = 231)	21% (*n* = 63)
Presence of National Antibiotic Guideline	78% (*n* = 229)	22% (*n* = 66)
Restriction on antibiotic use by pharmacists	72% (*n* = 211)	28% (*n* = 84)
Interactions between drug representative & doctors	65% (*n* = 192)	35% (*n* = 102)
Clinician’s preference for innovator antibiotic	49% (*n* = 144)	51% (*n* = 151)
Pressure from patients to prescribe antibiotic	48% (*n* = 143)	52% (*n* = 152)

Footnote: ASP—Antimicrobial Stewardship Program, ID—Infectious disease.

**Table 6:** Affecting the pharmacist’s recommendation of antibiotic choice.

Factors Affecting Pharmacist’s Recommendation	Always/Often	Never/Rarely/Sometimes
Variability in types and availability of antibiotics in hospital	81% (*n* = 234)	19% (*n* = 55)
Critically ill/immunocompromised patients	68% (*n* = 196)	32% (*n* = 94)
Risk of missing an infection	63% (*n* = 185)	37% (*n* = 108)
Toxicity profile of the particular antibiotic	63% (*n* = 183)	37% (*n* = 108)
The stock level of antibiotics	61% (*n* = 178)	39% (*n* = 116)
Risk of inducing resistant pathogens	51% (*n* = 149)	49% (*n* = 143)
Cost savings for hospital	32% (*n* = 93)	68% (*n* = 201)
Generic vs Innovator brand	15% (*n* = 44)	85% (*n* = 248)
